# Compact inertial sensors for measuring external disturbances of physics experiments

**DOI:** 10.1038/s41598-024-68623-0

**Published:** 2024-08-01

**Authors:** Jonathan J. Carter, Pascal Birckigt, Oliver Gerberding, Sina M. Koehlenbeck

**Affiliations:** 1 Max Planck Institute for Gravitational Physics (Albert Einstein Institute), Callinstr. 38, Hannover, Germany; 2 Institute for Gravitational Physics, Leibniz Universität Hannover, Callinstr. 38, Hannover, Germany; 3 Fraunhofer Institute for Applied Optics and Precision Engineering, Albert-Einstein-Str. 7, Jena, Germany; 4 Institut für Experimentalphysik, Universität Hamburg, Luruper Chaussee 149, Hamburg, Germany

**Keywords:** Optical sensors, Applied physics

## Abstract

Compact, high-precision inertial sensors are needed in the control schemes of many modern physics experiments to isolate them from disturbances caused by seismic motion. We present an inertial sensor whose mechanical oscillator fits on a one-inch diameter optic. The oscillators achieve a mechanical Quality factor of a fundamental oscillation mode of 600,000 and a resonance frequency of 50 Hz, giving them a suspension thermal noise floor lower than all commercially available inertial sensors. The motion of this fundamental mode is suitable to encode inertial motion into the sensor readout. The oscillator is combined with an optical resonator readout scheme that achieves a displacement noise of 100 fm/$\sqrt{\text{Hz}}$ above 0.2 Hz. We validate the sensors’ noise floor using a huddle test. Below 20 Hz, the sensor offers comparable performance to the best inertial sensors available today while being a fraction of the size. Above 20 Hz, the sensor is, to the author’s knowledge, the best demonstrated in the literature to date for such a sensor, with a self-noise floor of 0.1 n*g/*$\sqrt{\text{Hz}}$. The excellent performance of the sensors across seismically relevant frequencies, vacuum compatibility, and compact size make it a prime candidate for integration into sophisticated seismic isolation schemes, such as those used by gravitational wave detectors.

## Inertial sensors needed for physics experiments

Inertial sensors are used when we need to measure residual acceleration. They see wide use from spacecraft to mining exploration. However, physicists often use them alongside precise experiments^1–4^, which often have to contend with seismic activity disturbing their sensitive measurements. A key example of this is gravitational wave detectors such as the Laser Interferometer Gravitational Wave Observatories (LIGO)^5^, which use a suite of inertial sensors to achieve the levels of seismic isolation required to operate^1,6^.

High-precision inertial sensors are needed to isolate from seismic motion with active control schemes. To achieve a good performance, a traditional high-precision inertial sensor will use a large test mass, typically $\sim$kg^1,6^. Large sensors are bulky, limiting the locations around the experiments where they can be deployed. Compact inertial sensors exist in the form of Micro-Electro-Mechanical Systems sensors^7–11^. These sensors typically have $\upmu$g-ng of suspended test mass, but compensate for this with a high Mechanical Quality factor (Q factor) oscillation. Due to increased suspension thermal noise, the low test mass prevents them from reaching the same performance as the bulkier sensors.

Guzman et al. were the first to show a high Q oscillator with a gram scale mass^12,13^. The work showed that an oscillator design using two 100$\upmu$m thick bridges can support larger test masses without degrading the Q factor. Since then, various groups have begun evolution of the idea to adapt it for specific niches^14–19^. There is a strong desire to produce a design suitable for use inside the seismic isolation system of a gravitational wave detector. To contribute meaningfully to the seismic isolation control scheme, the sensors must have sensitivity comparable to current high-precision inertial sensors (for example, the Sercel L-4C (L-4C)).

This paper explores the complete design of a novel inertial sensor suitable for this task. A mechanical oscillator is designed, manufactured, and tested to show that it is suitable for use in the inertial sensor. The oscillator, which encompasses all the mechanical parts of the complete sensor, is designed to fit on a 1” diameter optic. This mechanical oscillator is combined with a Pound Drever Hall (PDH) locked^20^ 5 cm long optical resonator as a readout of the test mass position. We show a design with excellent sensitivity in a compact housing. The inertial sensor presented here is the first with such a high Q factor to be integrated with a high finesse optical resonator. At the same time, the oscillator achieves a m*Q* product of 2 tonnes, the highest of the Author’s Knowledge to date for a gram scale oscillator^17,19^. The self-noise of the device above 20 Hz is also the lowest available at the time of publishing^19,21,22^.

## Inertial sensor physics

A typical inertial sensor has two necessary parts. First, a means of suspending a test mass to remain inertially stable. Second, a means of reading out the distance between the suspended mass and the frame of reference. The relative motion of a suspended test mass, $\Delta X (f)$, to the absolute inertial motion of the system, $X_\mathrm{{g}}$, is given by

1 $$\begin{aligned} \frac{\Delta X (f)}{X_{\text {g}}(f)}=\frac{-f^2}{f^2-{f_0}^2-\frac{i{f_0}^2}{Q}}, \end{aligned}$$

As a function of frequency, *f*, where $f_0$ is the natural frequency of oscillation of the fundamental mode, and *Q* is the Mechanical Quality factor defined as

2 $$\begin{aligned} Q=2\pi \frac{\text {Energy Stored}}{\text {Energy Dissipated per Oscillation Cycle}}. \end{aligned}$$

Inertial sensors should be designed to minimise all spurious noise terms that can limit their sensitivity. The noises can be broken down into those that cause the test mass position to change without external force and those that cause us to mismeasure the position of the test mass.

Suspension thermal noise is the primary source of noise that disturbs the test mass position. It originates from the thermally-driven excitations of the molecular degrees of freedom of the test mass coupling to test mass motion through the fluctuation-dissipation theorem. It is often the fundamental limit of a design. Equations defining the limits of this noise source have been well defined in several places^23^. Suspension thermal noise typically becomes a problem for inertial sensors that target a sensitive bandwidth below 100 Hz^12,19,24^.

How thermal noise scales depends on the damping mechanism. When the damping is related to internal mechanical behaviour, it usually depends on displacement. This is called structural damping. The acceleration noise from structural damping is given by

3 $$\begin{aligned} \tilde{A}(f)=\sqrt{\frac{8\pi k_\mathrm{{b}}Tf_0^2}{ mf Q}} \end{aligned}$$

where $k_\mathrm{{b}}$ is the Boltzmann Constant and *T* is the temperature. The factors that make a low noise inertial sensor are already apparent. We need a high mass, low natural frequency, and high Q factor. Large inertial sensors achieve low damping losses using large proof masses with soft suspensions. Gram-scale inertial sensors must compensate for this mass loss to achieve high precision by using high Q factors, typically at least in the order of 10, 000^12,14, 19^. To build a high-precision inertial sensor, we must build an oscillator capable of achieving these high capable of achieving these high and integrate it with a readout method capable of measuring the motion of the suspended test mass.

### Encoding inertial motion

This work aims to produce an inertial sensor that could sit alongside other physics experiments, particularly optical ones. It was, therefore, decided to fit the entire mechanical part on the wafer of a standard optic, with 1” diameter and 1/4” thickness. As the overall aim of the work is to integrate such sensors inside gravitational wave observatories^5,25^, the sensor’s overall performance needed to be comparable to the high-performance sensors already used on site. This means of order n*g*/$\sqrt{\text{Hz}}$ sensitivity across all or part of the relevant control bandwidth (0.03-100 Hz)^1,6^. With similar performance to the current sensors used there, but in a compact package, we can add to the control schemes of such isolation by having sensors whose measurements better reflect the isolation requirements of the control scheme. This is because the sensors can be much better positioned in the readout scheme, closer to core parts that must be isolated. A control scheme can then focus on isolating this position. This is especially true for experiments in a vacuum, where many classical sensors must sit in a separate pressurised can, and for optical experiments, where such sensors can sit alongside the beam path.

The key element controlling the mechanical behaviour of the oscillator is the design of the thin bridges connecting the suspended test mass to the outer frame, which we call the flexures. They must ensure a soft suspension to keep the mass inertially still, leading to a low $f_0$, be low loss to ensure an excellent thermal noise floor, be sufficiently resilient to not break under the weight of the test mass, and be designed to push higher order modes to higher frequencies.

In a previous publication, the theoretical optimal design of gram scale resonators has been studied^26^. The paper considers the case of a gram scale resonator and how to optimise the design to achieve the best thermal noise in a resonator that does not fracture under operation. We used the results from this paper to design the resonator used in this work. To allow for handling and shipping, a maximum load of 5 *g* was used to estimate the upper load an oscillator should survive. The estimates on material dissipation depth, surface and bulk loss from^26^ were also used. The target was to achieve a noise performance comparable to the state-of-the-art inertial sensors used at LIGO. An initial study suggested that 50 Hz was a good target for $f_0$ as it would require flexures that still fit in the constraint of a one-inch optic and were not too long to be made. The thermal noise was then optimised using the conditions in^26^ to get the optimal design for these constraints. The result was that the flexures must be at least 22 mm long, 180*rmu*m thick, and a total of 4 mm high (which in this case could be split into 4 1 mm high flexures) while supporting 3 g of test mass (including bonded mirror). The oscillators were made of Corning 7890-0F fused silica due to its high purity and low bulk loss^27^. The expected Q factor from these oscillators was estimated as having contributions of Thermoelastic Damping (TED)^28^ and Material Loss^29^. The ${Q_\mathrm{{TED}}}^{-1}$ on resonance was estimated using a derivation from Ref.^30^ as ${1.5\times 10^-6}$. The material loss was slightly lower, with to an estimated ${Q_\mathrm{{Mat}}}^{-1}$ of ${5\times {10}^-7}$. We estimated an expected total Q factor of 500,000. A more comprehensive description of the oscillator design can be found in Ref.^26^.

**Figure 1:**
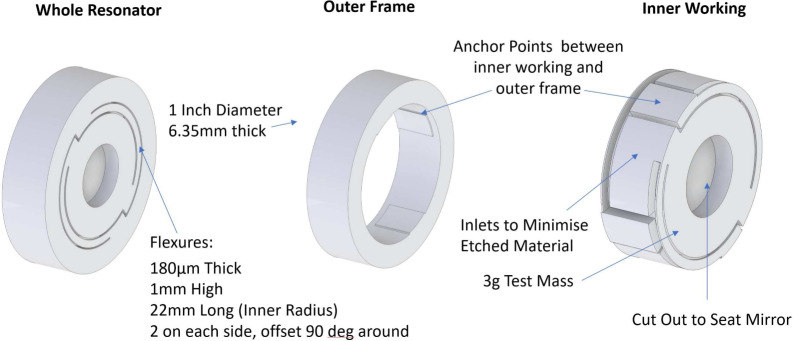
An annotated drawing of the key design features of the 50 Hz oscillator. The flexure parameters were designed to make the oscillator withstand 5 *g* of acceleration while providing a $f_0$ of 50 Hz . The second picture shows the outer frame and the third pannel the inner working of the oscillator. The material was carefully chosen to minimise the amount etched and prevent the flexures from contacting under maximum load. The inner radius of curvature was set at 0.1 mm on all corners to distribute the stress localised in these areas.

**Figure 2:**
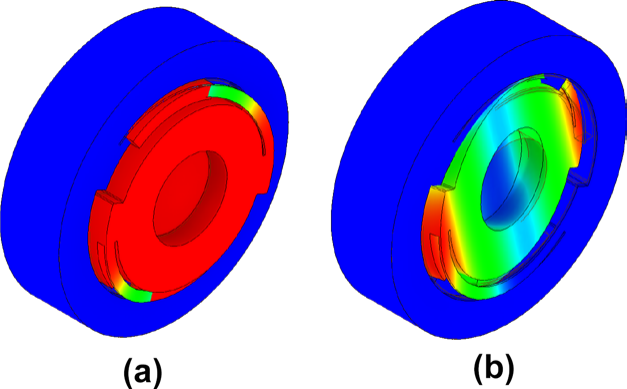
Fundamental and 1st order modes of the 50 Hz oscillator. The colour scale indicates the moving parts during oscillation, with red indicating more motion and blue remaining stationary in the sensor’s reference frame. The fundamental mode makes an in and out-of-plane oscillation similar to a corkscrew. This behaviour makes the resonator suitable for measuring motion in the direction of the optic plane. The 1st order mode defines the upper limit of the sensor’s sensitive bandwidth. Measurements at frequencies below the fundamental frequency are possible using Eq. (1), but a reduced sensitivity is achieved the further below resonance we go. (**a**) 50 Hz Fundamental “Corkscrew" Mode, (**b**) 209 Hz 1st Order “Tip-Tilt" Mode.

With the flexure geometry decided, they must fit in the overall geometry of the piece. The final result of this is shown in Fig. 1. The aim is to maximise the separation between the fundamental and higher-order modes while minimising the amount of material removed. Previous resonators of a similar geometry have used parallelogram structures to force the motion to be constrained mainly to one dimension^12,13, 17, 19^. Here, an alternating set of flexures in a disk creates a suspended test mass in the middle of the piece and a frame around the outside that can be clamped to the measurement frame of reference. The design results in a “corkscrew” mode of oscillation as the fundamental mode, as shown in Fig. 2. The mode travels directly in and out of the outer frame, meaning the sensor is largely only sensitive to oscillations from that degree of freedom. The next highest mode of oscillation is at 209 Hz where several “tip-tilt” modes occur.

The oscillators required the attachment of a high-reflectivity coating. Previous experience with an Ion Beam Sputtering (IBS) of Niobola/Fused Silica resulted in a significant degradation of Q factor. It was found that the attachment of small precoated mirrors with UV-cured Glue did not cause losses in Q factor. To accommodate this, a small cutout was made on the oscillator to seat the mirror. The cutout would prevent a mass imbalance between the two sides of the oscillator and allow for easy alignment.

## Building inertial sensors

The company FEMTOprint produced the oscillators^31^ using a laser-assisted wet chemical etching technique. With the sensors produced, they were tested to verify the simulation’s accuracy. Testing the sensors involved three steps: verifying the oscillator’s mechanical performance, the optical readout performance, and the sensor’s overall performance.

### Mechanical oscillator behaviour

Three parameters defined the mechanical behaviour of the oscillator: the suspended mass, $f_0$ and Q factor. A test mass of 3.1 g was estimated using 3D modelling and the density of the material. $f_0$ and Q factor were assessed by a ringdown experiment. A ringdown experiment involves exciting motion with a sharp impulse and tracking the decaying oscillation. The decay can be fit to an envelope of

4 $$\begin{aligned} A(t)=A_0\, \text{exp} \left( {-\frac{\pi f_0 t}{Q}}\right) , \end{aligned}$$

where *A* is the position at time *t*, and $A_0$ is the amplitude of oscillation at time $t=0$. The specific implementation of this ringdown setup is described in detail in Ref.^24^. The same heterodyne interferometer was used to track the motion of the test mass, with a piezo stack fixed to the base of the oscillator to excite motion. The experiment was isolated by passive isolation feet with a resonance frequency of 7 Hz to prevent residual ground vibrations from disturbing the measurement or exciting additional motion. Finally, the experiment took place in vacuum pressure of ${1\times {10}^-6}$bar to limit viscous damping effects from residual air pressure. $f_0$ was estimated by taking the amplitude spectral density of the signal and finding the bin with the highest peak. For oscillators with high Q factor. MATLAB’s nlinfit routine was used to extract the fit. The uncertainties quoted on measurements are reached from the covariance matrix on the fit. The first 10 minutes of data were not fitted to as higher order modes were also excited, and these were allowed to decay first to prevent spoiling the measurement.

**Figure 3:**
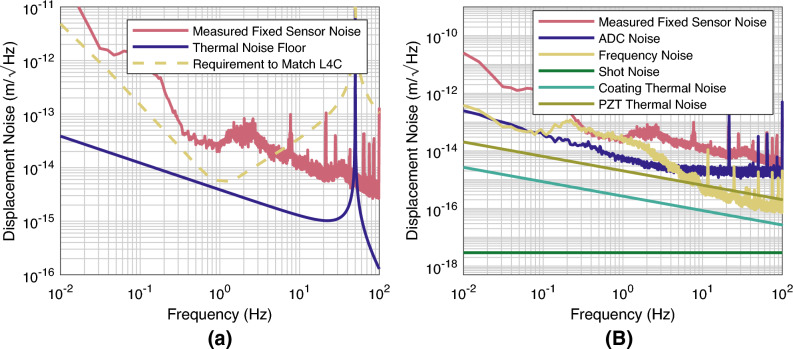
Assessment of the PDH locked optical resonators readout scheme when used with a 50 Hz oscillator. The fixed sensor noise is measured with a standard mirror included instead of a mechanical oscillator as the end mirror of the cavity. Hence, any noise here is related directly to the readout method. (**a**) shows the requirements of the readout to match both an L4C sensor and the thermal noise floor of the oscillator. The graph is shown in displacement noise, which is the actual motion of the test mass, and so does not account for the response of the suspension. The projection of the requirement to match an L4C is calculated by taking the performance of an L4C and inverting it with the transfer function in equation 1. (**b**) Shows the known noise sources of the readout. None explain well its limit over the whole range. Between 1 and 100 Hz the limit is thought to be linked to thermal expansion of the breadboard, but this has not been confirmed. Below 0.3 Hz, a wide variety of low-frequency noises could limit the readout, such as scattered light and polarisation-related effects. As they are common to both IIS in Fig. 5, there must be some common cause to this noise.

The oscillators achieved a mean $f_0$ of 50.3 Hz with a standard deviation of 0.4 Hz, and a mean Q factor of 637000, with a standard deviation of 18000. Each oscillator has a $f_0$ close to the target of 50 Hz and well within the tolerance from manufacture, which gave an expected $f_0$ range of ±8 Hz, suggesting the quoted tolerance of average flexure thickness $\pm 10$ $\mathrm {\mu }$m was overestimated. Each oscillator achieved a Q factor of over 600,000, higher than the initial estimate, indicating surface contamination was lower than expected. Regardless, these high Q factors mean that the sensors would have a sufficiently low thermal noise such that the thermal noise floor of the oscillators is below that of many of the state-of-the-art inertial sensors available today across a large bandwidth, shown by Fig. 3.

### Optical readout of test masses

With an excellent thermal noise floor, the next step is to read out the position of the test mass with sufficient precision. There is a plethora of compact precision readout schemes, both commercial and in recent publications^13,21,32–38^. In particular, cavity readouts have been shown to reach the thermal noise floor of micro-oscillators^39^. Figure 3a shows the requirement for a readout scheme combined with these oscillators to match the thermal noise, with readout noise. The choice of readout for an inertial sensor will depend on the designer’s experience with a specific technique, noise performance, and dynamic range of the readout compared to the expected motion of the test mass. For high Q factor sensors in seismically active environments, the large relative test mass motion will force the usage of a readout scheme with high dynamic range, such as heterodyne interferometry. As these sensors were aimed to operate in a seismically quiet environment, the solution chosen here was to use a Pound Drever Hall (PDH) locked optical resonator readout^20^ due to the ability of such schemes to achieve a low noise floor.

**Figure 4:**
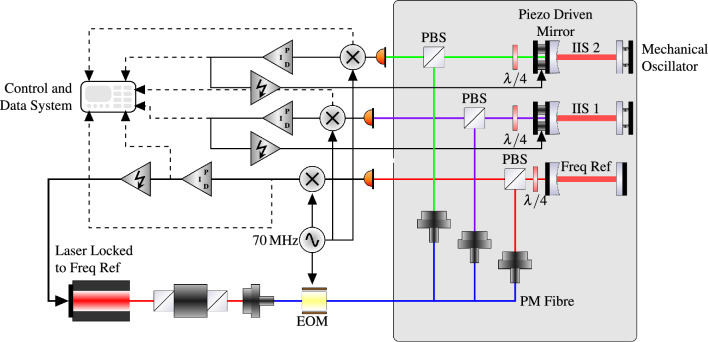
The layout of the huddle test measurement setup. Three sensors sat next to each other in vacuum. One frequency reference and two IIS. Each Sensor had an identical input and readout setup. Each sensors had approximately 4 mW of injected power, and where modulated with 70 MHz sidebands, which were demodulated in the photodetector. The frequency reference cavity was use as a frequency reference for the laser. Meanwhile the two OIS were held in lock by using mechanical Piezo Electric stacks mounted behind the in-coupling mirror to control the cavity length.

The schematic of this readout is shown in Fig. 4. The oscillator is one end mirror of an optical resonator. The out-of-plane motion of the fundamental mode of an oscillator changes the length of the optical cavity. The length change causes a change in the demodulated PDH signal. The cavity length must be controlled to keep the field in the cavity resonating. To do this, an active control loop was implemented in each resonator. A Piezo Electric Stack was mounted behind each in-coupling mirror of the Interferometric Inertial Sensor (IIS). The cavity length could be tuned to stay on resonance by controlling the voltage on the Piezo Electric Stack. The controls were implemented using an analogue servo. The control loops for the IIS had Unity Gain Frequency of $\sim$600 Hz. A further readout scheme was used on a rigid cavity, which was used as a frequency reference. The frequency reference used the same optical design but was kept locked by shifting the laser’s frequency. In addition, the mirrors were rigidly attached to an ultra-low expansion glass spacer to minimise thermal coupling to length changes in the cavity. The control loop of the frequency reference had two integrator stages, with a Unity Gain Frequency (UGF) of 20 kHz. The full parameters of the optical resonators can be found in Table 1.

**Table 1:** The parameters of the optical resonators used to read out test mass position in the inertial sensors.

Parameter	Value
Laser wavelength	1064 nm
Incoupling mirror reflectivity	98.5±0.4%
Oscillator coating reflectivity	99.4±0.4%
Incoupling mirror radius of curvature	0.25 m
Oscillator mirror radius of curvature	Flat
Cavity length	5 cm
Free spectral range	3 GHz
Line width	14 MHz
Finesse	312
Input power	4 mW

The overall size of the readout is controlled by the space needed to mode match the laser beams to the cavities. The implemented setup used an initially collimated beam from a commercial coupler and then a series of lenses over 50 cm of length to achieve the desired beam. A custom fibre injector with a mode designed to match the cavity could dramatically shorten the space needed to fit this to simply the fibre injector, a $\lambda /4$ waveplate, and the cavity. The PBS could optionally be included before the fiber, allowing the sensing head to be arbitrarily distanced from the readout electronics and photodiodes.

The performance of the optical readout was assessed by switching one of the oscillators in Fig. 4 with a rigid mirror of the same reflectivity. This allowed us to measure the sensor’s readout noise floor, shown in Fig. 3b). The limit of the readout is not clearly understood across a large section of the frequency range, although the figure shows that many of the fundamental limits are not limiting. The frequency noise is estimated as the error signal of the frequency reference cavity, the ADC (analogue to digitial) noise is measured by simply turning the laser off and measuring the resultant noise. The shot noise is estimated by calculations from^40^, while the other coating thermal noise is estimated with assumptions about the surface loss from^41^ about the tantalum coating of the mirrors, with the noise from this being calculated by the method described in Ref.^42^. Finally, the PZT thermal Noise was estimated by measuring the impedance of the piezo stack and using the fluctuation-dissipation theorem. The noise above 1 Hz is possibly caused by thermally induced length fluctuations in the base plates, though no temperature sensor available is able to confirm this. Below 0.5 Hz the noise takes a very different shape, as it will be shown later this noise is coherent between the sensors, at least in part, and so a common source is likely. However, at these frequencies, a wide variety of noise sources could prove to be the limit, such as scattered light, polarisation drifts in the fibers, relative motion between the PDs, which do not sit on the isolated breadboard, and the experiment.

Regardless, the readout scheme offers excellent sensitivity when compared to many others in recent literature^3,13,21,32–39, 43^. In addition, it is sufficient to achieve comparable performance to a L-4C across most of the bandwidth typically used for active seismic isolation (0.01-100 Hz).

## Evaluating inertial sensor performance

With the sensors designed and assembled, we wished to evaluate the sensor’s overall performance. To do this, a huddle test was performed^44^. Here, multiple sensors are clustered together with their sensitive axis aligned. The sensors can then subtract the coherent parts of their signal using a subtract the coherent parts of their signal using routine^45^. Doing so allows us to measure the true noise of the each of sensors, even when the measurements are polluted from residual vibrations disturbing the measurement. The whole setup in Fig. 4 was used to perform this experiment where two IIS were set up, along with a frequency reference. Ground-mounted L4Cs were also used for additional subtraction.

Even though we aim to take out the coherent motion of the sensors, it is still advisable to minimise the inertial motion of the sensors, as residual motion differences between the sensors will limit the measurement. In order to maximise the coherent motion of the sensors the whole experiment was built on a 1/2 ” thick stainless steel breadboard. The stiff breadboard would ensure common motion between the sensors. Three stages of isolation were used. Passive isolation feet were used on the optical table, which the vacuum sat upon. The vacuum also sat on a commercial active isolation stage. Finally, the breadboard in the vacuum tank sat on 4 isolation feet with a 7 Hz resonance frequency. Three L-4Cs in a Cartesian constellation were placed on the tank to compare their measurements to the IIS. These L-4Cs would also benefit from the first two isolation stages, but not the last passive in vacuum stage.

**Figure 5:**
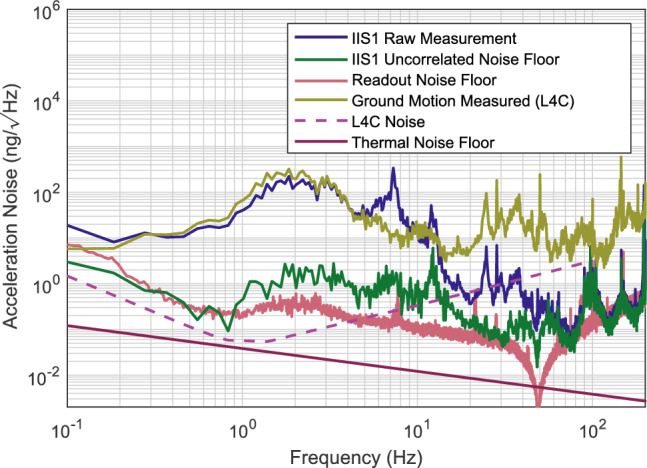
The results of the huddle test of two IIS with a frequency reference and ground mounted L-4Cs as a comparison. The readout noise floor is the readout measured in Fig. 3 projected into acceleration units. The yellow and blue curves show the raw measured signal from a geophone mounted on the vacuum tank and the IIS, respectively. These have good agreement below 7 Hz, showing the sensors are well calibrated. The IIS sits on a passive isolation stage with a resonance at 7 Hz, which causes the discrepancy in raw signals above this frequency. In green, the noise of the IIS signal after all coherent signals have been subtracted is shown. The self-noise of the IIS is limited by readout noise below 1 Hz and above 20 Hz. Between these values, the sensor’s measurement is likely limited by motion differences between the two sensors. The self noises of an L-4C and the suspension thermal noise floor are also plotted as a reference.

The experiment was set up, and measurements were taken overnight and at weekends to find seismically quiet data stretches. The measured noise from IIS1 is shown in Fig. 5. The pre-subtraction measurement agrees well with the noise of the L-4C below 7 Hz as we expect, showing the sensor is well calibrated. The result of the coherent subtraction is shown in green in this Figure. The sensor is limited by the displacement noise below 1 Hz. Below 1 Hz the sensors appear to perform better than the displacement sensitivity, suggesting a coherent noise source dominates the readout. This is not seen in the pure displacement readout, as only a single resonator was tested. Hence, a single inertial sensor would be limited by displacement readout in this range. From 1-20 Hz, the sensor is neither limited by readout nor thermal noise. As the shape is similar to that of the pre-subtraction signal, likely, the measured noise is still residual motion differences between the two sensors. Likely, this is due to slight differences in the sensitive axis of the two IIS. The sensors must be tested in a better-isolated environment to verify this. The device’s sensitivity is, therefore, still limited by the displacement sensitivity. At higher frequencies, several peaks are seen, the cause of which is uncertain, but could be related to higher order modes of oscillation down coupling into readout.

The IIS achieves a sub-n*g* performance across most of the control bandwidth of gravitational wave detectors (0.3-100 Hz), with the exception of a few peaks of measured noise. Above 20 Hz the sensor is one of the best available. The sensors’ testing environment limits the measured performance. The readout noise couldn’t be fully understood; therefore, the theoretical noise limit has not been optimised further. However, the digitisation noise limit can be overcome with appropriate analogue filters or a faster, lower noise analogue to digital converter. The thermal noise of this particular oscillator could then be reached. However, future developments in fused silica and cryogenic silicon oscillators might even surpass this sensor’s suspension thermal noise. The piezoelectric element thermal noise would then become a fundamental limit to this scheme and a future IIS would require construction without movable mirrors. Instead, one could use a high precision long-range read out^3,46^ or feedback on the laser frequency. The latter would require a high dynamic range frequency shifter or additional laser sources.

## Conclusions

By compacting down size of an inertial sensor onto a 1” optic without sacrificing the performance, we show a widely deployable sensor that can be used alongside a variety of physics experiments. By integrating the mechanical performance onto such a small device, groups can fit these sensors close to their sensitive experiments. Many groups interested in using these sensors would already have the expertise and equipment necessary to read out the test mass position with sufficient sensitivity to use such an inertial sensor in these experiments.

Overall, the two required pieces for an inertial sensor, a suspension and displacement sensor, are working, achieving outstanding performance for a sensor of such a small size. The performance of the sensors has been validated to be near its expected noise floor using a simple laboratory setup. Although the sensor does not quite reach the performance of larger inertial sensors at low frequencies, it has several advantages over any device available today. Its compact footprint means it can easily be deployed in many difficult-to-reach locations without unbalancing them. A fibre optic can be used to inject the laser beam into the sensor, meaning the compact sensor can be deployed far away from any data processing. The whole device is vacuum compatible and so can easily be deployed alongside many physics experiments without the usual need for a vault. Furthermore, as the device can operate in any orientation, multiple devices can be used to create 3D sensing in any arbitrary orientation. As interferometric readout is used the device is able to self calibrate.

Ultimately, adopting such sensors alongside larger physics experiments will allow us to isolate them from the scourge of residual vibration disturbances, allowing us better to probe many of the open questions in physics today (Supplementary Information).

### Supplementary Information

Supplementary Information.

## Data Availability

The data used in this study is available upon reasonable request to the authors.
