# Supplementary material for: Compact inertial sensors for measuring external disturbances of physics experiments
Source: Sci Rep. 2024 Aug 1;14:17775. doi: 10.1038/s41598-024-68623-0 (PMC11294580; doi:10.1038/s41598-024-68623-0)
Supplement: Supplementary file 1 — Supplementary Information. [file 41598_2024_68623_MOESM1_ESM.pdf]

Methods of Compact Inertial Sensors for Measuring External Disturbances of Physics Experiments.

Ringdown Experiment

A ringdown experiment is a common method of measuring an oscillator's Mechanical Quality factor (Q factor). To perform this experiment, the oscillator must be excited and its decaying oscillations measured. The time taken for the energy of the oscillation to fall to $e^{-2\pi}$ is recorded (remembering that energy is proportional to displacement squared). Multiplying by the oscillation frequency gives an approximate value for the Q factor as long as it is sufficiently large. For this experiment, we take the peak amplitude of each oscillation and fit these points to the function

$$A = A_0 e^{\left(-\frac{\pi f_0 t}{Q}\right)}, \tag{1}$$

where A is the amplitude of oscillation, f_0 is the natural frequency, and t is the time since excitation. The quality of the fit of the envelope to the data can be used to assess the measure of the Q factor. When there is a discrepancy, another mode of oscillation is likely causing a disturbance, or the signal decayed below measurement noise.

In order to perform a ringdown measurement of a mechanical oscillator, we need two things: a means of exciting motion and a means of measuring motion.

We achieved the former by mounting a large Piezo Electric Stack (PZT) behind the oscillator holder. A step voltage would be applied across the PZT, causing it to expand. The stack would expand into the holder of the oscillator driving it. A kick voltage of 20 V was sufficient to excite motion at about 1×10^{-7} m; enough to be easily seen above the interferometers noise floor. Supplementary Figure 1 shows the schematics of the interferometer used for this. The beam undergoes preparation in fibre outside the vacuum with the Acousto-Optic Modulators (AOMs). In the vacuum chamber, it is converted to free-beam via fibre collimators. We use two interferometers. The reference interferometer consists of only fixed optics. Meanwhile, one arm of the measurement interferometer includes the oscillator, which encodes its motion in the phase of the beat note. A Moku:Lab acting as a phasemeter reads out both arms, using IQ demodulation. The difference between the two interferometer phases subtracted the common motion to reduce the noise floor further. The kick from the PZT can excite other modes of oscillation in the mount and oscillator. In principle, these modes can be filtered out. However, waiting for these modes to decay before fitting the main oscillation is still advisable. Countermotion between oscillator modes and mount modes may lead to excess friction losses.

The oscillator must operate in a vacuum to prevent air pressure damping. A Q factor of 7000 was the highest measured in air in the prototype batch. The free beam optics are breadboard mounted to allow for quick switching between experiments in the vacuum and are shown in Supplementary Figure 2. Mounted on a breadboard outside the vacuum were the fibre optics. The breadboard sits upon four VIB100 isolation feet from the Newport Corporation to isolate from seismic disturbances. The experiment sits in a vacuum tank; the pre-pump was outside the lab to avoid additional disturbances. The measurements were conducted at a pressure of 1×10^{-7} mbar after 24 hours of pumping.

An example ringdown is shown in Supplementary Figure 3. The fundamental mode fits well with the exponential decay, showing we are well isolated from disturbances re-exciting the measurement during ringdown.

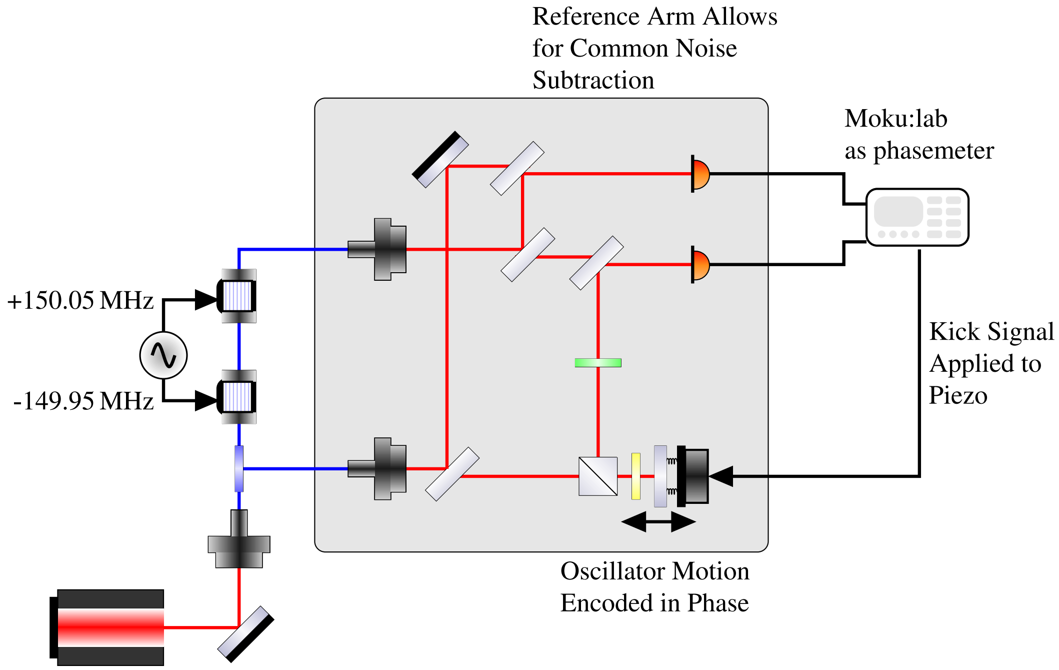

Supplementary Figure 1. The schematics of the ringdown experiment. The setup has two interferometers, both heterodyne, read out with a phasemeter. The one which does not contain the oscillator is used as a reference. The other is the measurement interferometer. All free beam beamsplitters are 50/50. Contrast can be maximised by matching the power in the two arms of the measurement interferometer using the appropriate fibre-splitting ratio, which must account for the sample's reflectivity. The Acousto-Optic Modulators (AOMs) have a combined efficiency of $\sim 25\%$. Fused silica has an uncoated reflectivity of $\sim 4\%$ at 1064 nm.

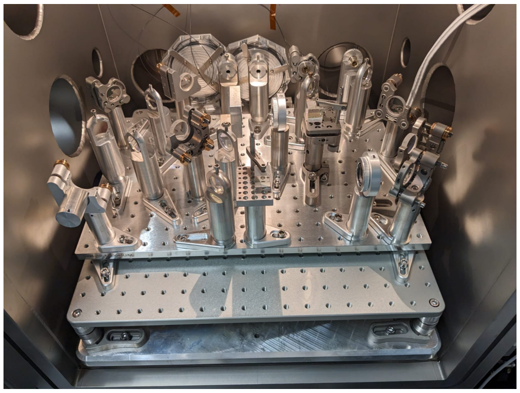

Supplementary Figure 2. A photo of the optical breadboard used in the ringdown measurement. A large base breadboard is attached to four vibrational isolation feet to keep the experiment isolated from excitation from the ground or pumps.

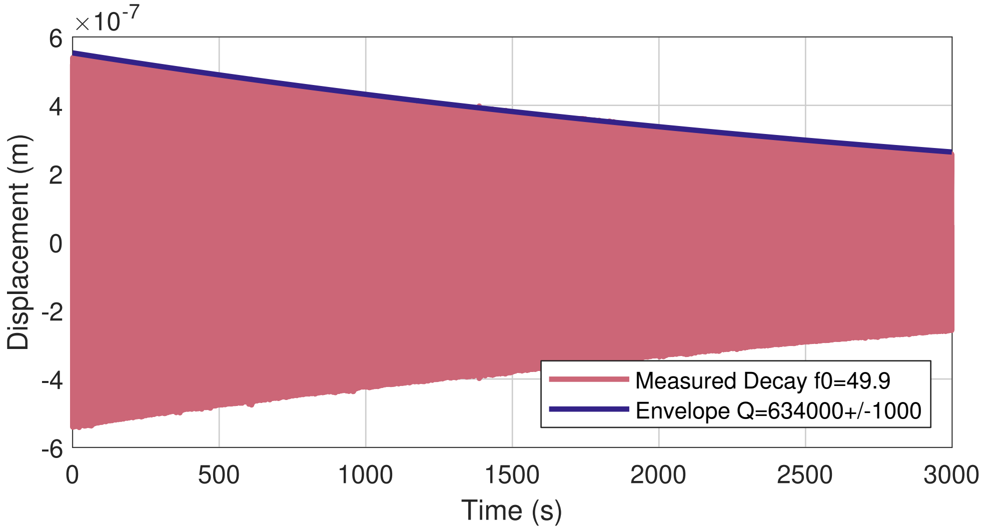

Supplementary Figure 3. An example ringdown of the oscillator showing a Q over 600,000.

Isolation and layout of huddle test breadboard

In Supplementary Figure 4 we show the IIS aligned on the breadboard. This breadboard sat on 4 Newport Corporation VIB100 feet, which offer passive isolation above 7 Hz. This, in turn, sat on a commercially available Accurian i4 stage from Park Systems. Finally, the whole system sat on a floating optical table.

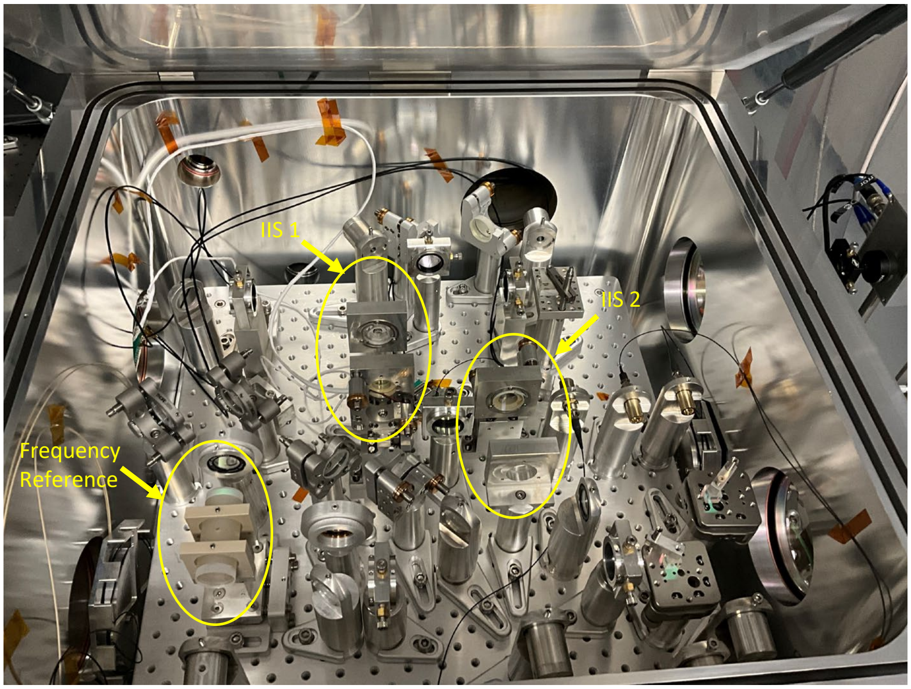

Supplementary Figure 4. An annotated photo of the breadboard used in the huddle test measurements. The two IIS were positioned close to each other. Photodetection was done through viewports on the right side of the photograph. The breadboard sits on three stages of isolation from outside disturbances, two passive and one active.
